# Fishing against the odds: fishers’ motivations to carry on fishing in the wake of the hindering EU Common Fishery Policy—a case study in North Shields, UK

**DOI:** 10.1007/s40152-021-00227-0

**Published:** 2021-06-26

**Authors:** Des Christy, Edwin B. P. de Jong, Luuk Knippenberg

**Affiliations:** Department of Anthropology and Development Studies, Radboud University Nijmegen, 6500 HE Nijmegen, The Netherlands

**Keywords:** Fishers, Motivation, Habitus, Common Fisheries Policy, North Shields, UK

## Abstract

The UK’s fishing industry has contracted considerably since 1972 due to overfishing, increased fuel prices, and implementation of the European Union (EU) Common Fisheries Policy (CFP). Despite this decline affecting the industry at large and the incomes of fishers, some fishers have carried on, or even freshly started or returned to the business. Why have these fishers done so despite the challenges they encounter in the fishing industry? In this article, we investigate why some fishers still choose to fish in the wake of all the EU regulations designed to control overfishing by reducing the size of the industry and discouraging entry by taking measures that affect revenues. Our data are collected through ethnographic research involving participant observation and interviews with fishers in North Shields, England. Based on our findings, we argue that the decision to carry on fishing, or even to return, is predominantly based on so-called intrinsic motivations, rather than on cost-benefit calculations, and stems from three interlinked basic human emotional needs which fishing seems to fulfil: the need to connect (sometimes also defined as the need to relate or belong); the desire for autonomy; and the desire to show competence (and have that competence recognized by relevant others). As such, the findings offer a fresh way to explain fishers’ decisions, based on a deliberated choice, to remain or leave the sector, and to understand and interrogate the challenges confronting present-day fishing both on a local level in the UK and also for Europe at large.

## Introduction

It is mid-April 2019, a pretty good time of the year for fishing. In North Shields, most of the boats have already been put to sea but Danny’s^1^ boat is still at the quay. His boat has a serious engine problem and needs to be repaired, which will take about 3 weeks. “Can you imagine? How much money I will lose in these three weeks?” Danny says to Greg, another fisher from North Shields. Like many other fishers in North Shields, he has seen a decrease in income. The last time he earned big money from fishing was back in the 1990s, when he made more than £30,000 a year. “Do not get me wrong, we still made £28,000–£29,000 last year but compared to 20 years ago this is nothing. You know what I mean?” He then explained, “well, you see, for us now, with the prawn boats, I am making a living, but that is about it. I am making enough money to pay my bills and maintain my boat, but I am not knocking doors out of windows [being really busy].” Despite the fact that he has not been able to increase his income over the past 20 years, he does not have any intention of leaving the job, no matter how difficult it is to keep going.

One condition set for the UK for joining the European Community (EC) in 1972 was that its fishers had to share “their” fishing waters with other member states. A parliamentary bill organizing this was hurriedly passed in 1970, before negotiations for the admittance of the UK to the EC started (Stenson and Gray 1995). A decade later, in 1983, the EC introduced the Common Fisheries Policy (CFP) that strictly demarcated and managed allocation and access to fishing for all member states (Churchill and Owen 2010). The CFP uses “relative stability” as a basic principle, based on historical catch data from the period 1973–1978 as a reference in making allocations (Oliver 1998). “Relative stability” was translated into the so-called total allowable catch (TAC) for each fish stock, divided between the member states, and fixed in the form of allocated national quotas (Hoefnagel et al. 2015). Each member state was free to determine how these national quotas were subdivided and allocated to fishers and the fishing fleet within its country, and how the quota attribution was to be regulated (Hatcher and Read 2001)

That the period of 1973–1978 was used as a point of reference for the fishing allocation was unfavorable for the UK. By the late 1970s, the UK fishing industry had lost access to the rich fishing grounds around Iceland due to the establishment of exclusive economic zones (EEZs) that allowed Iceland to claim a very large exclusive fishing zone (Phillipson and Symes 2001). In 1972, Iceland introduced a zone that stretched 50 miles offshore, and that zone was extended to 200 miles in 1975. British fishers were granted only limited and temporary fishing rights within this zone (Steinsson 2016). This led to a significant decline in British fish catches, which resulted in a far lower fish quota allocation under the CFP than expected.

Soon after the introduction of the CFP, new input controls were introduced through a licensing system in a further effort to limit the size and number of fishing boats. Prior to 1983, fishing licenses were only required for fishing boats of 40ft (12.19 m) and over in length, and these were fairly easy to acquire. With the introduction of the CFP, the requirement for fishing licenses was extended to all boats longer than 10 m and, in 1993, to boats of 10 m and less (Hatcher and Read 2001). Furthermore, in 1992, decommissioning schemes were introduced as the next step to reduce fishing capacity. In short, these fishing regulations heavily influenced and increasingly controlled the fishing industry by determining what kind of fish and how much could be caught, where fishing was allowed, and when, and by what types of boats from which countries. These EU regulations resulted in a steep decline in the fishing industry, i.e., the number of fishing boats, and the number of people employed. This process was accelerated by all kinds of technological improvements to the boats, fishing gear, storage, and fishing techniques, and through capital investments used to scale up and modernize the sector (Symes and Phillipson 2009). The number of fishers in England declined drastically from 14,442 in 1981 to 6250 in 2018 (Elliott and Holden 2019). All these developments, the strict application of the introduced regulations, but above all the perception that quotas were not allocated in a fair way, created a strong aversion among the remaining fishers to the CFP, and to the EU which was ultimately held responsible (Evans et al. 2018; Phillipson and Symes 2018). This is also offered as an explanation for why so many fishers in the UK voted in favor of Brexit and leaving the EU during the UK’s EU referendum (McAngus 2018; Phillipson and Symes 2018; Agnisola et al. 2019).

Danny’s story above is a good example of the impact of the regulations. They were in fact designed to restrict overfishing, by reducing the size of the industry, discouraging (re-) entry, and also by introducing measures intended to reduce revenues (Curtis and Jones 2016) (Tidd et al. 2011). To keep on fishing and make as much money as before, they had to invest and modernize, and above all secure a large enough quota of the right fish. This triggered a tough competition “for the survival of the fittest.”

Recent studies have offered further insight into the ways these processes worked, in the UK and elsewhere in the EU, and also show that they, often directly, or indirectly with the help of quota, fishing gear limitations, or other prescriptions, worked through revenue reduction to force fishers to leave the industry (Tidd et al. 2011; Johnsen and Vik 2013; Lagares and Ordaz 2015; White 2015). Lagares and Ordaz (2015) show, in their studies on the Spanish purse seine fishing fleet, that new limitations placed on the type of fishing gear had an immediate effect on revenues and led many fishers to decide to leave the industry. Fishers who used a single type of fishing gear and generated only small revenues were more likely to quit than those who operated with several types of gear and thus had more opportunities to fish and gain a greater income. Lagares and Ordaz concluded that decisions to leave the sector were mainly determined by revenue levels.

Their findings are in line with those of Tidd et al. (2011) concerning the reasons to leave or enter the industry among English North Sea beam trawler fishers (Tidd et al. 2011). These fishers tended to leave when profits started to drop below average incomes alongside, or because of, declining fish stock and increasing competition.

Johnsen and Vik (2013) mention, in the case of Norwegian fishers, also the importance of income and revenues. They identified a number of push and pull factors influencing fishers’ decisions to leave. Better opportunities, such as the possibility to enter higher education, or finding another job (offshore, onshore, or even abroad), were important pull factors. Important push factors were injuries that limited or prevented their possibilities to fish, having the boat impounded for violating fishing regulations or failing to meet payments to the bank, and the lack of a reliable and predictable income. Fishing had become an economically hazardous activity in terms of risks and revenues, while other more secure career opportunities had become more numerous and accessible.

It would seem, based on the above studies, that the drivers of fishers’ decisions to stay, leave, or (re-)enter are largely based on a rational assessment of costs against benefits, and especially economic benefits in terms of revenues. However, there are still many active fishers who, based on cost-benefit calculations, should have left. This shows that such decisions are not merely a result of cost-benefit calculations, or even purely rational choices. Many fishers determined to persevere, even though the economic odds were against them, new fishers even entered the industry, and some fishers returned. This leads to the question of why these fishers decided to stay, enter, and re-enter the fishing industry, against the odds and despite all the challenges and obstacles put in their way.

In 2011, the environmental social scientists Urquhart et al. (2011) appealed for a broadening of the scope of the study of fishing and fishers to include social and cultural aspects in order to gain a deeper understanding of the interaction between fishers and their communities, and of their social and psychological motivations to fish and keep on fishing, such as the role of identity and sense of place (p. 246). Their call inspired some social scientists (Nightingale 2013; Reed et al. 2013; Ross 2013; White 2015; Gustavsson et al. 2017; Gustavsson and Riley 2018a, b). Reed et al. (2013) indeed found that fishing deeply influenced the life and identity of fishers: that it is far more than just a job. White (2015) concluded that there are certain structural and relational mechanisms that hindered aspiring young fishers, especially those from non-fishing families, from entering the crab fisheries in Norfolk, UK. For youngsters to be able to access a job on a fishing boat, they either needed to be related (through family or friends) to the skipper or start fishing at another port to gain experience and savings, so that they could eventually become boat-owning skippers themselves. Gustavsson et al. (2017) stressed the need to adopt concepts that include but also exceed the economic aspects of fishing and the drivers of fishers to keep fishing (p. 105).

Therefore, in this article, we investigate the motivations of fishers to fish and to continue fishing notwithstanding the regulations designed to discourage them, and the fact that the revenues are often low, declining, or less than the salaries offered by other jobs, jobs they could also opt for, and perhaps would do if their decisions were based purely on an economic cost-benefit calculation. We will also look at the social and cultural contexts in which fishers are embedded. Hence, we look for explanations other than the common economic ones to explain the motivation for these fishers to continue or even to (re-)start fishing. We do so by applying the so-called self-determination theory (SDT) developed by the social psychologists Ryan and Deci, and also use the notion of habitus and doxa, developed by the sociologist Bourdieu to deepen the explanation.

## Theoretically framing a self-determined fisher

Since the social psychologists Ryan and Deci first developed their so-called self-determination theory (SDT) in the 1980s, they have consistently argued and convincingly shown that motivations that make people act are intrinsic, i.e., internal, based on a sense of inherent interest and joy, and not, or at least far less, because of extrinsic stimuli, such as the promise of financial rewards or profit. In their later studies, they even show and explain how extrinsic stimuli have to be “translated” into intrinsic motivations in order to make people act.

Based on extensive research, Ryan and Deci argue that there are three core psychological needs that all people, independent of their culture, share that have to be enabled (stimulated, fulfilled, or answered) to give a person a sense of purpose and a feeling of well-being, in other words, to be motivated, to act, and/or to see purpose in an action (Deci et al. 1989; Deci and Ryan 2000; Ryan and Deci 2000a, b; Deci and Ryan 2008; Ryan and Deci 2017). These three core needs are competence, relatedness, and autonomy. The notions of competence and autonomy refer to the idea of self-determination, the idea that people need to have the feeling that they do have a choice, that they themselves can decide—at least to a large extent—what to do or not to do. Autonomy and competence are both inward- as well as outward-oriented needs, in other words personally as well as socially shaped. One needs to perceive oneself as autonomous and competent, and others have to perceive them as such.

To be self-determined means to experience a sense of choice, of autonomy and competence, in initiating and regulating one’s actions. The feeling of competence is the thrill of undertaking a physical and/or mental challenge and then meeting it. A feeling of competence can be gained, for example, through positive feedback, meeting challenges, freedom from demeaning evaluations, and communication. To increase perceived competence, a sense of autonomy is necessary. This sense of autonomy is required to self-organize and regulate one’s behavior. The notion of relatedness, the third core psychological need, refers to the fact that we as individuals are first and foremost social beings. We need other people, we long to belong, we need to relate to function, to feel good, to feel a purpose, and to act. According to Ryan and Deci, it is the need to relate, and the way we do that, that makes us people. As described above, personal feelings of competence and autonomy depend on the presence and appreciation of “the other.” Such feelings are developed in relation to the other, and have to be perceived and preferably, but not necessarily, valued by related (and appreciated) others. Relatedness is about attachments and feelings of security, belongingness, and intimacy with, and care for, others (Deci et al. 1989; Deci and Ryan 2000; Ryan and Deci 2000a, b; Deci and Ryan 2008; Ryan and Deci 2017).

These insights of Ryan and Deci can serve to understand why fishers make choices that are not always understandable if we perceive them from purely a cost-benefit perspective. However, we need to remain aware, as Ryan and Deci themselves acknowledge, that extrinsic motivations, such as the desire for an external reward, although far less stimulating (or motivating) than intrinsic motivation, are socially important. The possibility of being autonomous is curtailed by all kinds of social demands and roles that require individuals to assume responsibility for often intrinsically uninteresting tasks. It is often here that the need to relate overrules the need for autonomy (Ryan and Deci 2000a).

We have seen that Ryan and Deci’s ideas can be helpful in explaining the deeper motivations and choices made by the fishers given that their actions cannot always be explained by referring to rational choice theory based on cost-benefit calculations. Nevertheless, there is another aspect that needs to be explained in order to fully understand the motivations and choices of the fishers, an aspect that is not sufficiently addressed by SDT. That is, that the “choices” they make, and the practices they employ, even their autonomous acts, always take place within a certain range of activities that are considered acceptable, or “normal,” where other choices that could also have been made are not. This partly individual, partly collective, aspect of behavior cannot be explained by SDT theory.

In addressing this aspect of choice and behavior, we use the ideas of Bourdieu (1977, 1990) on habitus and doxa. The French sociologist Bourdieu introduced these two concepts to explain why people behave in a certain way and make certain choices (although Bourdieu would not approve of the word choice in this context) and think this is logical, if not the only possible or normal way to behave or chose, whereas in reality other behaviors or choices would have been possible, and often more logical.

The explanation of Bourdieu is that people and groups have individually and collectively internalized certain “practices,” respectively called habitus for an individual and doxa for groups, and have done this so deeply and totally that they no longer even reflect on them. They see these practices as an integral aspect of who they are, as familiar and indispensable as, for instance, an arm or leg.

People, individually and collectively, mostly act in a certain way without thinking and only start to think about it if that way of acting encounters an obstacle that cannot be overcome by “doing what they are used to do.” If this happens, if the effect of the “internalized practice,” the habitus or doxa as Bourdieu calls it, is paralyzed or blocked, it becomes visible as what it is, i.e., a specific “normalized” practice, an internalized way of acting, behaving, and perceiving (determining what is normal and what is not). Then, it can suddenly become clear that not only are other practices possible, but sometimes even better ones. A shorthand for explaining the working of habitus and doxa is the expression “fish don’t talk about the water, until it is no longer there.”

We use the notions of habitus and doxa to argue that the practices of the fishers, and their “choices,” in SDT terminology and in their own words, have to be understood as being embedded in an almost unchanging web of partly individually internalized (habitus) and partly collectively externalized (doxa) conditions. In the words of Bourdieu: “the product of a particular class of objective regularities, that generate ‘reasonable’ or ‘common-sense’, behavior, possible within the limits of these regularities, and positively sanctioned because they are objectively adjusted to the logic characteristic field, whose objective future they anticipated” (Bourdieu 1990). In other words, we are talking about practices and choices considered to be “normal” and “logical” (or possible, acceptable, achievable, inspirable) by the actors themselves and their social environment.

In the remainder of this article, we investigate the motivations of the fishers in North Shields by applying self-determination theory, enriched with the notions of habitus and doxa, as outlined above, as the frame of analysis.

## Methodology

The data on which this study is based were collected in three periods, roughly totaling 12 months, of ethnographic fieldwork, preceded by some preliminary research. The preliminary research (October to November 2017) was conducted in two locations, Lowestoft and North Shields. The “specific reasons” as Yin (2003, 2009) calls it for selecting these locations was their long history of fishing as well as administering large fleets of vessels. In the 2016 UK fisheries report, these two ports hosted more registered fishing boats than anywhere else in eastern England (Richardson 2017). Despite the report stating that there were 294 vessels registered in Lowestoft in 2016, we faced difficulties during the preliminary research in finding “active fishers.”

Meanwhile, North Shields, based on the 2016 report, had 369 registered vessels but, in reality, it appeared that there were only around 30 active local fishing boats docked at North Shields Fish Quay. During our interview with the North Shields’ harbor master, we found out that vessels registered at the harbor do not necessarily dock at North Shields. The same caveat applies to figures for the number of fishers. According to the official 2018 government figures, 462 fishers are registered in North Shields Elliott and Holden (2019) but apparently several of them are fishing out of other ports for most of the year. In reality, the figure is closer to about 70 fishers (28 boats operated by a skipper and 1–4 crew plus 2 boats with just a skipper).^2^

**Fig. 1:**
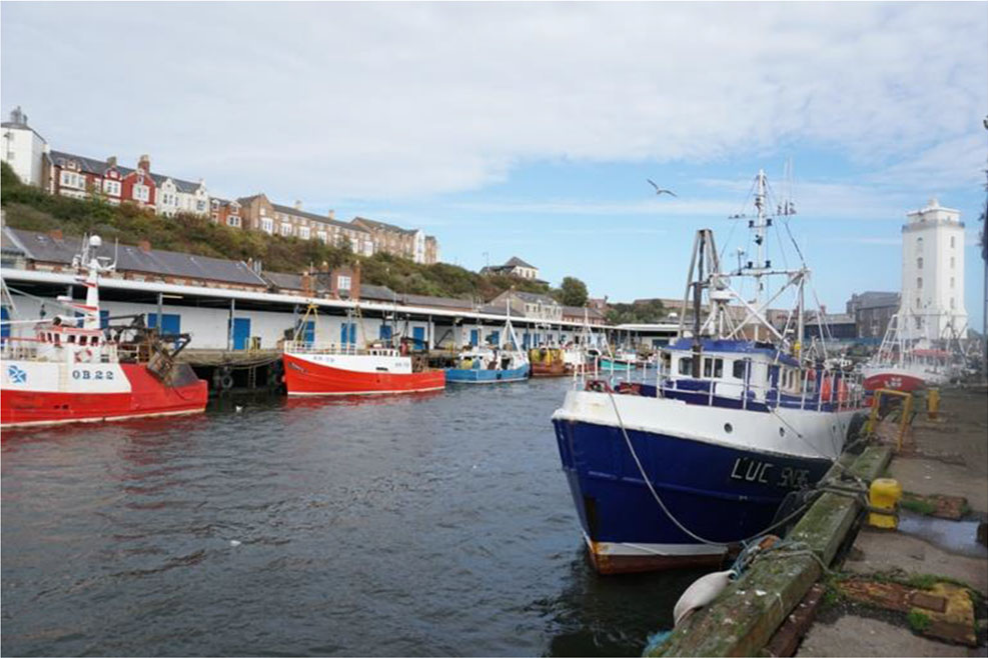
North Shields Fish Quay October 2018

Furthermore, we located two active organizations of retired fishers (with 12 and 30 members), who were easy to approach and provided a lot of background information. Some members of these organizations also introduced us to active fishers. From this exploratory investigation, we concluded that there was far more fishing activity in North Shields than in Lowestoft. Hence, we selected North Shields as our case study location for conducting ethnographic fieldwork between June 2018 and March 2020 (Fig. 1).

Nadel-Klein (2003) emphasized that ethnographic investigation is needed if one is to properly understand the consequences of public attitudes, policies, national agendas, and transnational economic forces for localities, and therefore, in our case, to understand fishers’ motivations and to position their experiences in the present day as well as in a historical context. To understand fishers’ experiences, we collected data through interviews (semi-structured and open) and conducted participant observations among active and retired fishers, meaning that we incorporate “small talk” Johnsen and Vik (2013) and “deep hanging out” (Geertz 1998) as important methodological tools. As such, data for this article were collected through various semi-structured in-depth, oral history and open interviews, small talk, and observations, often repeatedly engaged in with 16 active fishers and 3 retired fishers. The age of the interviewed fishers ranged from 24 to 73 years. The active fishers included eight boat-owning skippers, four skippers, and four crew members. These informants were selected through a combination of purposive and snowball sampling. The sampling was purposive in the sense that we tried to cover a representative number of fishers divided over generations, crew, skippers, and boat-owning skippers as well as retired fishers, but we also used snowball sampling to find and approach fishers. Prior to the first interviews, we asked the informants for their consent.

Most of the semi-structured in-depth interviews were conducted during the first meetings. These interviews were conducted on the basis of an interview guide. Afterwards, we mostly carried out more informal, oral history and open interviews to allow the fishers to share their life stories and experiences in the way and in the situations that felt most comfortable to them. These interviews were generally conducted in one of three settings: inside their boats, at the dock, or in the pubs. On a rare occasion, we also joined fishers on their fishing trip and joined in the fishing activities. When the fishers were not at sea, they spent most of their time at the quay, busy with onshore activities such as boat repairing and net mending. This appeared to be a good time to talk with them and join in the activity of net mending. Pubs became an important setting for not only talking and interviewing the fishers but also to observe their behavior and interactions with others as it was at these hostelries that fishers spent their free time and relaxed with their fellow fishers.

In addition, we also joined the weekly meetings of the retired fishers. There were two distinct meetings that we regularly attended: the ones held every Monday in the Fishermen’s Mission office, with 12 members; and the ones organized on Tuesdays in the community center, with around 30 members including both retired fishers and some retired mariners. Semi-structured, open, and oral history interviews with retired fishers were also conducted during these meetings. From these meetings and interviews with retired fishers, we collected information about fishers’ experiences in the past, and saw that most of them had started fishing during the 1960s. This information also helps in putting our data in a historical perspective.

The qualitative data, including field notes, photographs, interview data, and interview reports, were analyzed using emergent category designation (Erlandson et al. 1993). The data analysis, or, as Schensul and LeCompte (1999) phrase it, the process of systematically searching and arranging the interview transcripts, field notes, and other material to reduce them to a more manageable form that allows the researchers to tell a story about the people that is the focus of their research, consisted of three stages. In the first stage, all the materials were read to obtain an understanding of the main topics and themes, sorted and loosely coded accordingly, a process labeled “deconstruction” in the terminology of Corbin and Strauss (1990). In the second stage, the material was sorted into meaningful categories and domains, labeled, i.e., “construction” in the terms of Corbin and Strauss. The third and final stage, or “confirmation” according to Corbin and Strauss (1990), involved selective coding in which we integrated and refined the theory. During this phase, we looked selectively for cases that illustrated themes, both in terms of similarities and contrasts.

## Fishing from North Shields

Historical records of fishing activities in North Shields go back to 1225, when the port was built by the Tynemouth Priory. In 1820, the first fish market was constructed in the fishing town, followed by completion of the Union Quay in 1823. The industrialization of fishing in North Shields started when Purdy, a local businessman, used a steam tug to fish, and by doing so caught more fish than the other boats (Lawrence 2016). His success was noted by other fishers, also beyond North Shields, who started to adopt his ideas. In 1880, North Shields started to attract fishing boats from further afield, increasing the number of boats in the harbor considerably. In 1909, there were 76 trawlers based at North Shields that together caught 20,000 tons of herrings that year (Rowland 1994).

North Shields, is ideally located for North Sea fishing, with nearby fishing grounds considered to be among the richest in the North Sea (CDP 1978).^3^ A retired fisher, during an interview, remarked that there were only three options for a job after finishing school in his time: work in a coal mine, enter the army, or go fishing. “Although,” he added, “if someone was smart enough, he might have had a chance to find work in a ship building company.” For many youngsters of his generation, fishing was the preferred choice, no qualifications were needed, and more money was earned than in mining or the army. The halcyon days of deep-sea trawlers in North Shields came to a sudden end in the early 1970s because of the cod wars with Iceland. Subsequently, more and more smaller fishing boats started to operate at the expense of the larger boats and, in 1975, there were no longer any boats left over 42 m in length.^4^ The majority (81%) of the boats were between 12 and 24 m in length.

Currently, the roughly 30 fishing boats docked at North Shields are small to medium in size (up to 20 m in length), using trawl nets to catch prawns. Nowadays, North Shields is famous for landing prawns^5^. In 2019, the fishers of North Shields landed 2254 tonnes of prawns, or about 54% of the total prawns landed in England, with a total value of more than £7 million^6^. With most fishers now targeting mainly prawns, winter has become the busiest time for fishing activities in North Shields. In the peak season, there are also visiting boats, registered in Scotland, that fish from North Shields. Meanwhile, during the summer, most fishing boats will leave the North Shields Fish Quay and start fishing from the east (e.g., Eyemouth, Buckie) or west coasts (e.g., Mallaig) of Scotland.

Most of the fishing boats are family-owned businesses, operated by 1–4 people including the skipper. Some of the boat owners also work as the skipper on their own boat. Of all the local boats, there was only one not owned by a family business, but owned by Caley Fisheries, a Scottish fishing and offshore business. However, after our research, at the end of 2020, a group of local businessmen purchased the North Shields–based operations of Caley Fisheries, including the building and fish processing facility on the fish quay, and changed its name to 55 Fisheries. This company provides vessel management, chandlery, fish processing, and fish sales services to fishers. For small-scale fishers, like those in North Shields, working with such a fishing agency provides the support necessary to ensure that they can land and sell their fish and dock their boats.

## Fishing from North Shields under European Fisheries Regulations

The introduction of the EU CFP in 1983 deepened and accelerated the industry’s change and altered its nature. The whole nature of fishing was transformed, with major consequences for the incomes, autonomy, competences, and relatedness of the fishers including those between skippers and crew.

Three things were always brought up when we asked fishers in North Shields about the EU CFP: the struggle to obtain and retain a fishing license, the fish quota allocation, and the decommissioning program. The fishing license rules limit the total number of vessels, their size and power, and the extent to which one can switch between fish stocks and fishing methods. The fishing license determines the quota a fisher can catch in terms of the tonnage of fish they may land, while the quota allocation determines what type of fish can be caught, how much, and where.

When the licensing system was introduced, the UK government offered fishing licenses free of charge to active fishers. Prior to 1995, fishing licenses and quotas were non-transferable and attached to a boat, meaning that if a fisher lost his boat (by accident or otherwise) he also lost his quota rights. However, in 1995, the fishing license became detached from the boat, and the license formerly allocated to a specific vessel to catch a quota could be transferred from one owner to another.

Meanwhile, a decommissioning scheme was established in an effort to reduce fishing through reducing the number of fishing boats. The government offered payments to owners for scrapping their fishing boats. The program had the intended effect, but seriously harmed the North Shields fishing industry in terms of money, autonomy, and inter-relationships. Many skippers and fishers lost their job without any compensation, and it was not uncommon for fishing boat owners to hold on to their quotas even if their boats were withdrawn. Once a quota became detached from the fishing license and a boat, the boat owners could generate income by scrapping their fishing boats and renting out their quota rights. Some even accumulated fish quota, a group who became known as fireside or armchair fishers.

In response to the accumulated effects of all these measures, many fishers left the sector, but others in North Shields started, around the late 1990s, to focus on prawn fishing. When asked why, they commonly offered four reasons for this shift away from mixed fishing. They found that it became increasingly difficult to fish without getting fined or incurring additional quota costs when they landed fish at the quay. Second, valuable fish such as cod became difficult to catch in inshore waters. Third, they saw that the market price for prawns was quite attractive given the low quota costs. Moreover, it appeared that fishing boats less than 10 m long were entitled to catch around 20–30 tonnes of prawn every 3 months, which was far more tonnage than allowed with finfish species (see Table 1).

**Table 1:** UK regulations for catch limits in the North Sea for fishing boats 10 m and under, December 2019

Stock (species)	Period	Catch limits (tonnes)
Cod	Monthly	3
Haddock	Monthly	4
Hake	Monthly	1
Lemons and witches	Monthly	3
Sole	Monthly	4
Ling	Monthly	0.2
Nephrops (prawns)	Quarterly	30
Skates and rays	Monthly	4

However, switching to catching prawns required changes in many aspects of fishing. Some old fishers who used to work on the deep-sea trawlers, disparagingly call fishing for prawns “Mickey Mouse fishing.” Others, still active fishers, especially those who used to work on mixed fishing boats, say that fishing for prawns is not as exciting as “catching mixed species.” Danny for instance told us: “You turn lazy when fishing for prawns, because you keep returning to the same places, while fish move to different places. Then, you sometimes got big hauls, and true excitement.”

Fishing for prawns also meant that fishers began to rely heavily on markets outside the UK, and this is one of the most significant fish species exported. The main export destinations for prawns are France, Spain, and Italy^7^. During the COVID-19 outbreak, the export market became disrupted, and many distributors stopped buying prawns from the North Shields’ fishers. Some fishers decided to temporarily stop fishing, while others have started to sell their prawns directly to local outlets. As Andy, a boat-owning skipper, explained, “The problem with COVID-19 [i.e., knock-on effects], is that a lot of buyers [distributors] have closed their doors, so we have had to start selling our prawns to the public. But, even while the public is supporting us during these hard times, we are only able to fish as little as once a week.”

The downsizing of the fishing industry that started long before COVID-19 has also led to difficulties in recruiting competent crew. This has had an impact on the autonomy of the skippers and boat-owning skippers, and their relationship with their crews. Few youngsters are willing to work under the uncertain conditions of modern fishing. Skippers and boat-owning skippers have less choice, and as a result less freedom. They often have to accept anyone willing to work, and just wait to see whether the new shipmates prove capable or not. Boat-owning skippers, like Danny for example, admitted that they would give a chance to anyone that wanted the job: “You have got to give young lads a chance, whether they are good or not at sea, because they are going to be our future.”

Furthermore, the lack of a steady supply of crew has also changed skippers’ preferences towards crew. Even if a skipper or boat-owning skipper finds out that a new crew member is not up to the job, he continues to employ him, afraid of not finding a replacement or only an even worse one. Greg (65), who was a skipper on his own boat when we first met him but had since retired in 2018 due to health issues, commented: “You can still fire crew members but there is no guarantee that you will find anyone better. There is a chance that you would even get worse crew than what you had.”

Most of the fishing boats in North Shields apply a shared reward system for local crew members. Under this shared system, after deducting costs to cover expenses, the total income earned on a fishing trip will be divided with 50% going to the boat owner^8^ and 50% shared between the skipper and crew members^9^. New crew members, who are undergoing training, usually only get a half share until they prove fully capable of independently doing the job. This sharing system creates considerable uncertainty, as the crew could earn a lot on good trips but get nothing when the weather is bad and the boat stays in the harbor. Furthermore, the sharing system puts skippers and crews in similar positions as they are reliant on each other for achieving a good catch and the associated income.

In the past, a lot of young people wanted to enter the industry and work with respected skippers, those who were able to make money and keep the crew safe at the same time. Respected skippers could choose the best crew members since many fishers wanted to work with the best skippers since that would ensure a higher income. Hence, a good skipper at that time was often described by our informants as a “semi-god” who was able to instruct the company that owned the boat about the kind of crew members he wanted to work with. Ryan, a retired fisher who used to work on deep-sea trawlers operated by the Purdy Company, explained that “If someone got into any trouble with the skipper, and the skipper sacked him, he would not get another job because the skipper would inform the company about the matter and the company would inform other skippers. [So,] he would not get any job around the quay for weeks up to about six months. Until he learnt his lesson! Don’t ever argue with the skipper.”

Today’s skippers, no matter how good they are, have to adjust to the limited pool of available crew members. Skippers and boat-owning skippers therefore try to keep their good crew members through various means. Nowadays, a good skipper does not only have to be capable of finding spots where good quantities of prawns can be caught, they also need to work together with their crew on the deck (instead of staying in the wheelhouse), treat them like friends (lending them money, occasionally having drinks together, anticipating their needs).

## Reasons to (re-)start fishing

In the literature, fishing is often described as one of the most dangerous occupations due to the large physical and socioeconomic uncertainties (Acheson 1981; Ginkel Rob 2009; Symes et al. 2015). At the same time, that danger appears also to be a challenge and the attraction of fishing, at least for some fishers. Fishing out at sea with large waves can provide, for some, an intoxicating cocktail of challenge, competence, and freedom. Many fishers were born and raised in an environment where fishing was an enduring part of their childhood. Their families, neighbors, and friends were fishers, and the fish quay was their playground. Their aspiration was to become a fisher, and their motivation to continue fishing was not merely the money, but because that is “what they were, and what they valued.” This can be seen in Tom’s (a retired fisher) statement: “I was born and raised near the sea. So, every day, since I was a kid, I saw all the boats on the River Tyne from our window. So, you know, I grew up like that. If I was born a countryside boy, then my dream would have been of a farmer. But I was born next to the river.”

During the 1970s and early 1980s, the fishing sector was still economically attractive for many youngsters in North Shields. William is a good example. He was born and raised in North Shields, is the third generation of a fishing family, and used to play on the fishing quay as a child. He became a full-time fisher in 1975, when he was 15 years old, and got his skipper’s ticket 3 years later. William started fishing because that was what his family did, that was what he wanted, and that was what he could do best.

However, there are other fishers who had a real possibility to pursue other careers, but still decided to become fishers. Ron’s story is an example. Ron, a skipper and owner of a boat that is over 10 m in length, is part of the eighth generation within a fishing family. He always knew, since he was a child, that he wanted to be a fisher. However, his mother wanted him to find another job because some of his friends had lost their lives at sea. Ron listened, went to college, and finally qualified as an engineer. However, once he graduated, he told his mother “Now mother, after I’ve done what you want me to do, I will start to do what I want to do.” He went to the sea and started a fishing career as a trainee deckhand and moved up to become a full deckhand within 1 year. After 10 years of fishing, he obtained his skipper’s ticket.

In North Shields, we also found fishers who used to fish during the 1970s, quit their job because of insufficient income, but returned to fishing a couple of decades later. A good example of this is Jake, a skipper and owner of a boat under 10 m in length. He started to go down to the quay when he was still a boy to help fishers. After he finished school, he became a fisher (like William and many others). However, around 2006, when the incomes from fishing were rapidly decreasing, and rules and administration obligations were mounting, he decided to leave fishing and started working for an offshore company. In 2018, having become tired of working far away from home, Jake bought a small fishing boat and restarted fishing from North Shields. He returned to fishing, not only because he could, since he knew the business, but mainly because this offered him something he truly valued.

This was not only true for Jake but also for various other fishers that we interviewed. Ted (71) had also decided to become a full-time skipper once he had sufficient money to buy a boat. As with Jake, many highly appreciate the freedom offered by fishing, especially if they are a skipper and boat owner because then they are their own boss. They can decide where, when, and in what way to fish. They are free to do what they want to do, and they have the competence and the connections to do so.

Nowadays, there still are youngsters who become fishers. Some of them do it for the money (an extrinsic reason). That is quite understandable since there is considerable youth unemployment and the demand for crew members on fishing boats is still high, albeit less than in the “golden years.” These youngsters are mostly not born into a fishing family, but they are in networks (friends, cousins, neighbors) that have introduced them to fishing life. Other youngsters have more intrinsic reasons. These are mostly born into a fishing family, could do something else, but nevertheless have decided to become fishers. Some of them already knew that they wanted to become a fisher when they were still in school.

However, among this final category of youngsters, intrinsic and extrinsic reasons seem to be intertwined, as with Keith (24), a young skipper, who has been fishing since 2013. Keith started fishing because he could not get any other job after he had finished school. However, a more important factor in his decision was that he had two cousins and a great uncle who already worked as fishers. His two cousins offered him the job.

From the above discussion, we can conclude that intrinsic motivations (and habitus) are an important driver for people’s decision to start fishing. Their entire socioeconomic environment breathed fishing (it was their doxa), and their whole being was filled by it, both physically and mentally (and it solidified in their habitus), which fitted with their desire for autonomy, competence, and connectedness. This and the other illustrations are good examples of the importance and interrelatedness of intrinsic motivations, such as autonomy, competence, and relatedness, with habitus and doxa. Ron’s story is a good illustration of the power of habitus and even of doxa, but also of intrinsic motivation, in fact of the power of the combination of habitus and intrinsic motivation. Ron had always wanted to be a fisher since he was very young. That drive was most probably largely an internalized result of the fact that he was born into a fishing family, raised in a fishing environment, and surrounded by fishers, fishing stories, and fishing practices. The decision to listen to his mother’s advice was in this instance probably more extrinsic than intrinsic, even though it came from his own mother: he did what he was supposed to do as a good son, but it was not in tune with what had become his habitus. That is the likely explanation for why he decided to become a fisher only after he had finished his education, even though he had opportunities to work in a different sector with his engineering qualification.

The roles of autonomy, competence, and habitus are virtually self-evident in the above-mentioned cases. This is not the case with doxa. Doxa is crucial because choosing to become a fisher is not an obvious choice if it is not seen as normal or socially accepted. This was nicely expressed above by Tom while referring to the fact that he had grown up as a “river boy.” For younger generations, extrinsic motivation (such as being unable to find another job, or acceptance by the skipper) also appeared to be an important trigger, often intertwined with intrinsic motivation (such as a feeling of connectedness through networks). However, as many fishers noted, it is easy to enter the fishing industry, the real challenge is to endure and survive in the job. We now therefore discuss fishers’ motivations to stay in fishing.

## Reasons to stay

### Extrinsic motivation

For some fishers, their decision to stay in the industry was most likely driven by an extrinsic motivation: they were afraid of losing their income. Younger fishers, especially those that had already started a family, mentioned their obligations to pay the bills and the costs of raising their children, and that this drove them to stay in fishing. For them, fishing seems a far more certain future than looking for another job and starting again from scratch. For example, Toby (26) who has two daughters is working as a crew member on a fishing boat and has been fishing since he left school. He told us that he will stay in the job because he has no other qualifications than being a fisherman and he did not want to start from zero again. Keith’s situation is quite similar, as he explained: “Aye, it is quite hard to get another job. I just got stuck with it, because I got this job in fishing and now I am completely tied to it. I do really look for something else, but it is a struggle: to get something else and at the same time have the bills paid, if you know what I mean.”

Older fishers often mentioned that they still have a good income, although not as good as two decades ago. They like to refer to the highest income recently received. Adam, for example, a 58-year-old boat-owning skipper, mentioned that he once got almost £10,000 for 6 days of fishing. However, incomes in fishing are very unstable and range from sky high to virtually nothing: “on one trip, one can ‘hit the jackpot’ but, on another, the catch may barely cover the expenses of the trip.”

As described earlier, the EU’s efforts to reduce and control fishing and limit the catch led many fishers to quit. Tom, for example, after 15 years of fishing had to find another job in the 1980s because his income from fishing was no longer sufficient for a decent living. He started to work as a truck driver. However, Tom is also a good example of the strength of intrinsic motivations, in this case autonomy. While discussing his departure, he stated “I really liked it, you know. You are the man on the sea, just on your own.” He eventually stopped fishing because “this is just the way it went, everybody had to stop fishing. The European regulations force us to do so. Many fishers had to quit fishing at that time.” Here, we have a good example of a fisher who had an intrinsic drive to stay but forced out by other factors.

### Intrinsic motivation

“One cannot go fishing just because of the money, you have to like it” Robin (53), a crew member, said about what it means to be a fisher. Robin’s father had started to take him to the quay when he was 6 years old. He became a full-time fisher after he finished school despite his father not wanting him to do so. Robin was born and brought up in Scarborough but moved to North Shields 15 years ago to work as a crew member on a fishing boat. The fish quay and fishing boat are his home. If he is not at sea, he can easily be found on the fish quay whatever the weather. We asked him what his drive was to stay in fishing. He responded that fishing is more than just a job, “I meet nice people, they are my family, and this (fish quay) is my home.”

#### Relatedness

Robin’s story above is an example of a fisher deciding to stay in fishing for more than extrinsic motivations, in this case the desire to feel connected, one of the three core intrinsic motivations. Being connected means that he feels safe in the environment that he comprehends as family. His remarks are not that surprising. Fishing crews have to work in relatively small spaces on a fishing boat, and their work requires them to trust each other. “In fishing, one has to trust other crew members. It is about working together and trusting each other to get the work done,” Robin explained. Older fishers who once worked on larger fishing boats mentioned their close relationships with shipmates. For some fishers, this closeness is still maintained. Greg, for example, will always hire his two ex-shipmates to help him repair his fishing nets. When we asked how much he has to pay for their help, he commented “With them, I just pay a little, only for pocket money. But, when I land with my catch, they can take any fish they want from my boat.”

Albeit in a somewhat different way, connectedness is also offered as an explanation by today’s fishers. Gerald, who is skippering a boat that is over 10 m long, explained that it is necessary to have crew members who are close to each other as they have to share relatively small working spaces. That closeness also counts in the relationship between crew and skipper, given the already mentioned limited choice of crew members. Skippers nowadays also work on the deck, jointly with their crew, and the income is shared equally, creating greater equality between crew and skippers. Today, the skipper and crew drink together in the pub, something that rarely occurred in the past.

The language that fishers, both skippers and crew, use to communicate on the boats as well as onshore also creates a kind of relatedness. Rowdy language and banter is common but, as Robin explained, “fishers will always, when we can, do that stuff [banter and cursing] but we also know when it has to stop.” Max, a retired fisher, noted that fishing is the only job in which one can curse the boss without the boss feeling offended.

Skippers connect with each other through information exchange, for instance about fishing locations and the volume of prawns caught. This sharing is based on trust and reciprocity. Skippers share such information only with other skippers they trust and who they expect to provide similar information in return in the near future. They form, what they themselves call a small clique, based on this type of reciprocity principles. Skipper Eddie (40), for example, shares this kind of information with two other skippers, one his former skipper. Being part of a clique gives them a sense of security because anyone in the group who catches a lot of fish will tell the others where the fish can be found. The building of trust among clique members takes many interactions over a long period of time. These interactions are highly valued. Some skippers even mentioned that they do not need any help from other people to do their job but do depend on this exchange of information with other skippers. This fits well with Acheson’s (1981) arguments about the strategic importance of this form of agreement to reduce uncertainty, and the fact that these “agreements” can only be achieved if there is trust built through past interactions and common experiences.

To a large extent, the good relationships with other skippers have been developed because of the dangerous nature of fishing. Fishers realize that there will come a time when they are at sea and need help from other fishers. Doug, a skipper-boat owner, illustrated this well when he told us about the death of his son, Dylan, in August 2010. At that time, Dylan was on a fishing boat together with his oldest brother, when the boat collided with a cruise ship near Eyemouth. His brother, the skipper, was saved by other fishers working nearby, but Dylan could not be spotted. Massive search and rescue operations followed involving local authorities and about 20 local fishing boats for 2 days. After the official rescue operation was called off, local fishers were still helping Doug’s family find their missing son. Dylan’s body was eventually found by local fishers 3 months after the accident.

The above discussion shows how the feeling of relatedness was built through shared fishing experiences, mutual trust, as well as through the dangerous nature of fishing. However, this will never be achieved if one’s disposition does not fit with the nature of fishing.

#### Autonomy

The desire for freedom (or autonomy in Ryan and Deci’s phraseology) appeared to be one of the major factors in fishers’ decisions to remain in fishing. For most fishers, freedom is associated with being a man of the sea, part of nature, away from their responsibilities at home. Furthermore, freedom for some boat-owning skippers was also related to autonomy with regard to their fishing schedule. Matt, a 31-year-old skipper on a 17-m boat owned by his father, mentioned this explicitly “Let us just say that if I want to go to sea, I’ll go to sea. If I don’t want to go to the sea, I won’t go. You know I like boats, so I like to go out sailing. It is as simple as that.” Another example was provided by Ron: “I make my own decisions, and I do whatever I want. I am not controlled by anyone else. That is why it is a very good job, and I have a lot of freedom.” A similar example of freedom was provided by Ted. For him, the fishing job gives him the freedom to decide on which day, at what time, and how he will go fishing. These views are in line with the explanation by van Ginkel Rob (2001) that fishers valued their autonomy and the corresponding identity as an “independent individualist.”

The crew, since they have to follow the skipper’s schedule and orders, are more likely to associate the feeling of freedom with being a man of the sea, being part of nature. However, the fishers who highly appreciate the freedom to make decisions are usually those who have had an opportunity to become something other than a fisher. Similar to Ron’s situation, Matt’s father, Doug, was also a successful fisher but he did not want his son to follow in his footsteps and encouraged him to go to college instead. However, Matt had already decided when he was very young to become a fisher. Meanwhile, Ted, although his father was a trader, was raised in a fishing community and had relatives working as fishers. By the time he was 22, he already owned a boat but only used it part-time because he also worked for a local consultancy company. It was some time before he decided to become a full-time fisher.

For the fishers discussed above, being a fisher has been a deliberate choice, and their decision to remain in fishing is strongly related to the fact that their fishing life is not just a job but a way of living and part of their life. Here, we see how choices allow a greater sense of freedom for fishers. Their past circumstances (that structured their habitus) shape their decision to choose this way of life that gives them a sense of freedom.

#### Competence

The feeling of competence was also both explicitly and implicitly expressed by other fishers who stayed in the industry. They did this when describing what makes a successful skipper. Doug, for instance, explained that a successful skipper is one, just like him, who can generate enough income to give their crew a good share of the takings. “If the skipper looks after the boat, the boat will make you money” as Doug explained while showing his recent payment from the fishing agency^10^. A sense of competence was also expressed by Adam, a 57-year-old boat-owning skipper who fishes alone. When we asked him what skills fishers needed to have success, his answer was ambition: “I’m 57 now, and not many fishers at my age are still able to work alone. In fact, I don’t know anybody else who works alone with this kind of boat [over 10 meters].” To be a good skipper, one has to have many competences: the abilities to find the right fishing grounds to maximize profit, to take good care of the boat, to carry the correct and well-maintained fishing gear, and to manage the crew by carefully balancing between dividing tasks up in a directive and effective way and being their peer at the same time. These qualities cannot be achieved through following a fishing course, one has to go to sea for years to learn fishing in practice.

Locating the right fishing grounds can only be learnt through years of experience and sometimes with the help of other skippers in one’s clique. Finding the right ground involves not only knowing the spots where large numbers of prawns are to be found but also being able to factor in the distances to get there. As Greg explained: “If you can catch something within 10 miles, right, you can get by on half as much as what you can catch [if you were to travel for] 100 miles. Given the price of fuel and the time to go 100 miles at sea and return, we are speaking about a day, so the fuel will be expensive. If you sail for 10 miles, then it is an hour and half, then you are hauling in fish and getting back to the harbor, so with little fuel, you don’t need to catch so much.”

The skill and knowledge in finding fishing spots is intertwined with connectedness and is a necessary competence when it comes to targeting prawns. Some fishers explained that prawns are funny creatures, that their movement patterns differ from those of white or flat fish. This made the building of trust and cliques essential for fishers, and this requires good social skills. As noted by Matt, “at the end of the day, people will know if someone has told a lie or not.”

The skippers also mentioned that having good competences also offers an advantage at the fishing agency that manages their sales. Skippers who catch more fish than others often receive praise from their fishing agent. Moreover, the fishing agency in North Shields is more likely to give a loan to a skipper who has a good catch record. Such competences are more linked to extrinsic motivations, or external rewards, than to intrinsic drivers.

To summarize, the mastery of fishing cannot be achieved in a fishing school or through a few days of experience on a boat. As several fishers in North Shields regularly stated, a person who wants to learn fishing needs to jump on a boat and see whether they have sea legs or not. A person who has been to a fishing school might only last a few weeks on a fishing boat. New crew members are expected to adapt themselves and learn the skills of fishing as fast as they can. Otherwise, they will be negatively evaluated, which becomes visible through receiving smaller shares and the way other fishers think and talk about them. Meanwhile, positive feedback can be perceived in the form of a full share of the income, additional bonuses, and also by being praised by the skipper.

#### Hybrid motivation or identified regulation

It is not always possible to make a clear division between extrinsic and intrinsic motivation. We met fishers who started fishing for the money but then started to gain satisfaction from fishing. Many fishers started immediately after finishing school, and thus had no other qualifications to fall back upon. Their fishing competence had developed over a lengthy period and, as a result, matured and reached an advanced level, becoming part of their habitus. Hence, their initially extrinsic motivation became intertwined with an intrinsic motivation, as made clear by Ron who on the one hand said that he was too old to do anything else, but at the same time emphasized that he has specific competences related to fishing that were sufficient to make him decide to stay in the fishing industry.

Keith, who we introduced earlier, provided another good example. He is a skipper on one of the smaller boats, under 10 m long and owned by Doug. His first week in the industry was a nightmare, but he survived and stayed fishing because he needed the money. He tried to find something else but felt forced to persevere in order to be able to pay his bills. After 6 years of fishing, he has now reached a position where he projects his future as not only a skipper but also a boat owner. He is saving to buy his own boat, similar to the one he is currently working on. Keith’s story shows how extrinsic motivation can become internalized, a process that Ryan and Deci (2000) described as *identified regulation.* This is a process of internalization in which the subject starts to value the activity they do, and accept it as their own (in Keith’s case by projecting his future as a fisher who owns a fishing boat). Identified regulation is a good example of nurturing habitus (Murphy and Costa 2015). Keith started fishing because he could not get another job but, after 6 years of fishing, he has started to see himself as a fisher, and his future as skippering his own boat.

## Conclusions

Most of the existing studies that point to fishers taking a cost-benefit approach to decision-making, acting as a kind of “homo economicus” and choosing on rational grounds to leave or stay in the sector, do not provide a helpful framework for understanding why not all the fishers left the fishing industry in the wake of the hindering EU fishing regulations.

We have therefore sought other, more complete, explanations for why some fishers are motivated to continue or even (re-)start fishing by using Ryan and Deci’s self-determination theory and considering Bourdieu’s notions of habitus and doxa. Building on these ideas, we concluded that, alongside extrinsic, largely financial, motivations, there were intrinsic motivations that evolved from three basic and interlinked emotional needs that fishing seemed to fulfil: the needs to connect, or relatedness, to feel free, or autonomy, and to feel competent. It seems to be these intrinsic needs that predominantly explain why North Shield’s fishers carry on fishing against all the odds. The practices engaged in to achieve relatedness, autonomy, and competence are to an extent individually internalized and partly collectively externalized.

However, it is not always possible to divide the extrinsic and intrinsic motivations of North Shields’ fishers along clear lines. Sometimes, the initial extrinsic motivation becomes intertwined with an intrinsic motivation, with some fishers who started to fish simply for the money then beginning to gain satisfaction from fishing.

In conclusion, this study has sought a deeper understanding of the motivations of fishers in North Shields to carry on or even return to fishing despite the difficult times. In so doing, we encourage a more differentiated mode of thinking about the fishing industry, and fishers, by placing greater emphasis on human needs and values, while not ignoring the contexts and conditions in which they are embedded and the trends that put these values under pressure. This could contribute to more inclusive policymaking for the fishing industry that not only takes into account, but also anticipates, the people that are at the core of this sector in order to develop a broader policy that can help achieve a sustainable fishing industry.

This recommendation goes a step further than the advice seen elsewhere to introduce co-management, a step which incidentally the fishery sector already took some time ago, for instance through the 2009 introduction of the Association of Inshore Fisheries and Conservation Authorities (IFCAs) in England as successors to the Sea Fisheries Committees, some of which were established in the late 19^th^ century (see, for example, Rodwell et al. 2014). What we are encouraging is that, alongside attention to ecological and economic aspects of sustainability, as witnessed in the IFCAs and other measures taken in the past 15 years, also more attention should be paid to social aspects of sustainability. In the first place, this should address the intrinsic social motivations of fishers to quit fishing or not, and to adapt their fishing methods or not, factors we argue can be encompassed and explained by using the SDT theory of Deci and Ryan and the habitus and doxa concepts advocated by Bourdieu.

## Footnotes

1 All the informants in this article are given pseudonyms to protect the privacy of the individuals described.

2 In the winter months, around 50 vessels with an average crew size of 4–5 will visit the harbor. So, over the course of a year, around 250–300 fishers will visit North Shields, but they will not all be there at one time.

3 There was a short period between 1965 and 1973 where deep-sea fishing activities took place from North Shields.

4 Sea Fisheries Statistical Tables 1975. Accessed from: https://webarchive.nationalarchives.gov.uk/20140508034354/http://www.marinemanagement.org.uk/fisheries/statistics/annual_archive.htm

5 *Nephrops norvegicus*, known variously as the Norway lobster, Dublin Bay prawn, langoustine. We adopt the term “prawn” as used by the local fishers.

6 UK sea fisheries annual statistic report 2019. Accessed from https://www.gov.uk/government/statistics/uk-sea-fisheries-annual-statistics-report-2019

7 https://www.seafish.org/media/publications/T4_UK_export_trade_and_markets_for_selected_shellfish_species.pdf

8 The boat share covers the long-term maintenance costs (e.g., for new nets, or if the engine requires repair).

9 The sharing proportions may differ. If the boat is not owned by the skipper, the boat-share proportion might be higher to give the boat owner a share.

10 The fishing agency manages the catch and other administration for fishing boats. Most boat owners work with the fishing agency because it can guarantee that the boat can enter any fishing port. The agency also deals with the sale of the catch, as well as preparing all the essentials for fishing trips.
